# Supplementary material for: Geometric learning of knot topology
Source: Soft Matter. 2023 Oct 20;20(1):71–8. doi: 10.1039/d3sm01199b (PMC10732224; doi:10.1039/d3sm01199b)
Supplement: SM-020-D3SM01199B-s001 [file SM-020-D3SM01199B-s001.pdf]

Electronic Supplementary Material (ESI) for Soft Matter.
This journal is © The Royal Society of Chemistry 2023

Geometric Learning of Knot Topology: Supplementary Information

Joseph Lahoud Sleiman,[1,*] Filippo Conforto,[1,*] Yair Gutierrez Fosado,[1] and Davide Michieletto[1,2,†]

[1] *School of Physics and Astronomy, University of Edinburgh, Peter Guthrie Tait Road, Edinburgh, EH9 3FD, UK*

[2] *MRC Human Genetics Unit, Institute of Genetics and Cancer, University of Edinburgh, Edinburgh EH4 2XU, UK*

SIMULATION DETAILS

We model knotted curves as semi-flexible coarse-grained bead-spring polymers with $N = 100$ (unless otherwise stated) beads of size σ. The beads interact with each other via a purely repulsive Lennard-Jones potential,

$$U_{\rm LJ}(r) = \begin{cases} 4\epsilon\left[\left(\frac{\sigma}{r}\right)^{12} - \left(\frac{\sigma}{r}\right)^{6} + \frac{1}{4}\right] & r \leq r_c \\ 0 & r > r_c \end{cases}, \quad (1)$$

where r denotes the separation between the beads and the cut-off $r_c = 2^{1/6}\sigma$ is chosen so that only the repulsive part of the potential is used. Nearest-neighbour monomers along the contour of the chains are connected by finitely extensible nonlinear elastic (FENE) springs as,

$$U_{\rm FENE}(r) = \begin{cases} -0.5kR_0^2 \ln\left(1 - (r/R_0)^2\right) & r \leq R_0 \\ \infty & r > R_0 \end{cases}, \quad (2)$$

where $k = 30\epsilon/\sigma^2$ is the spring constant and $R_0 = 1.5\sigma$ is the maximum extension of the elastic FENE bond. This choice of potentials and parameters is essential to preclude thermally-driven strand crossings and therefore ensures that the global topology is preserved at all times [1]. Finally, we add bending rigidity via a Kratky-Porod potential, $U_{\rm bend}(\theta) = k_\theta\left(1 - \cos\theta\right)$, where θ is the angle formed between consecutive bonds and $k_\theta = 10k_BT$ is the bending constant, thus yielding a persistence length $l_p = 10\sigma$ (different values of l_p are considered below). Each bead's motion is then evolved via the Langevin equation

$$m\frac{dv_i}{dt} = -\gamma v_i - \nabla U + \sqrt{2k_BT\gamma}\eta \quad (3)$$

along each Cartesian component. Here, γ is the friction coefficient, m the mass of the bead, U the sum of the potentials acting on bead i and $\sqrt{2k_BT\gamma}\eta$ a noise term that obeys the fluctuation-dissipation theorem. The numerical evolution of the Langevin equation is done with a velocity-Verlet scheme with $dt = 0.01\tau_{LJ} = 0.01\sigma\sqrt{m/\epsilon}$ in LAMMPS [2]. In order to initialise the knotted chains, we use the ideal spatial representation used by KnotPlot and smoothly deform the contour to introduce LJ and FENE interactions before any strand-crossing occurs.

Sampling

To ensure that the conformations are uncorrelated and suitable for training our model, we evaluated the interval between samples via the auto-correlation time, τ_0. This characteristic time was calculated using the autocorrelation of the radius of gyration against time, τ i.e.

$$G(\tau) = \frac{\sum_t (R_g(t) - \langle R_g\rangle)(R_g(t+\tau) - \langle R_g\rangle)}{\sum_t (R_g(t) - \langle R_g\rangle)^2} \quad (4)$$

where $\langle R_g\rangle$ is the mean radius of gyration and $R_g(t)$ is the radius of gyration at time t. We then fitted an exponential decay with characteristic time τ_0 to measure the auto-correlation time. For a 100-bead knot, $\tau_0 \simeq 14\,10^3$ LAMMPS steps (not shown), and we thus collected 10^5 conformations every 10^5 steps, ensuring uncorrelated samples.

DATA PREPROCESSING

Feature Engineering

To train the NN, we label the polymer conformations with their topology, independently verified using Kymoknot [3, 4]. We also process the raw LAMMPS output to obtain geometric representations that are fed as input into the NNs. More specifically, we here consider 6 representations, defined as follows:

- **Cartesian (XYZ) coordinates**: we consider the 3D coordinates of each monomer along the chain, and to ensure that the absolute position of the polymer in space does not affect the model, we shift the polymer's centre of mass (CoM) to the origin.
- **Adjacent monomer distances**: we calculate the Euclidean distance between beads as $\boldsymbol{d}_i = \boldsymbol{r}_{i+1} - \boldsymbol{r}_i$ and normalise the obtained values between $[-1:1]$.
- **Local polymer curvature**: we calculate the local curvature along the polymer as

$$\Gamma(i) = \frac{1}{n}\sum_{j=i-n/2}^{i+n/2} \arccos\left(\frac{\boldsymbol{t}_{j-1,j}\cdot\boldsymbol{t}_{j,j+1}}{|\boldsymbol{t}_{j-1,j}||\boldsymbol{t}_{j,j+1}|}\right) \quad (5)$$

where $\boldsymbol{t}_{j,j+1} \equiv \boldsymbol{r}_{j+1} - \boldsymbol{r}_j$ is the tangent vector at bead j and $n = 20$ an averaging window.

- **Local bead density**: we calculate the local curvature as

$$\Delta(i) = \frac{1}{NV_R} \sum_{j \neq i}^{N} \Theta(R - |\boldsymbol{r}_i - \boldsymbol{r}_j|) \quad (6)$$

where $\Theta(x) = 1$ if $x > 0$ and 0 otherwise. In this equation, $V_R = 4\pi R^3/3$ is the volume of a sphere of radius R, and we take $R = 3\sigma$, in simulation units. The results obtained are broadly unchanged as long as this threshold is larger than the bead size but smaller than the entire polymer knot.

- **Local or 1D (unsigned) writhe**: we numerically compute the local crossing number using a generalisation of the better known global average crossing number [5], which we can write as [6]

$$\omega_{1D}(i) = \frac{2}{4\pi} \sum_{k=i-l_w}^{i} \sum_{l=i}^{i+l_w} \frac{|(\boldsymbol{t}_{k,k+1} \times \boldsymbol{t}_{l,l+1}) \cdot (\boldsymbol{r}_k - \boldsymbol{r}_l)|}{|\boldsymbol{r}_k - \boldsymbol{r}_l|^3} \quad (7)$$

where $l_w = 10$ is the window length over which the calculation is performed. In this equation, local self-crossings are the major contributing factors. A straight, non-self-crossing segment centred at bead i would yield $\omega_{1D}(i) = 0$. The maxima of this function along the chain indicate regions of maximum *local* self-entanglement. This quantity has been previously used to identify supercoiled plectonemes in simulated DNA [7, 8], branches in ring polymers [6] and self-entanglements in proteins [9].

- **Segment-to-All (StA) writhe**: we numerically compute the global or non-local crossing number by

$$\omega_{StA}(i) = \frac{2}{4\pi} \sum_{k=i-\frac{l_w}{2}}^{i+\frac{l_w}{2}} \sum_{l=0}^{N} \frac{(\boldsymbol{t}_{k,k+1} \times \boldsymbol{t}_{l,l+1}) \cdot (\boldsymbol{r}_k - \boldsymbol{r}_l)}{|\boldsymbol{r}_k - \boldsymbol{r}_l|^3} \quad (8)$$

which measures the entanglement of a polymer segment centred at bead i against the rest of the polymer contour. Eq. (8) is a generalisation of Eq. (7) where we do not restrict the calculation of the average crossing number to occur between contiguous polymer segments.

- **Segment-to-Segment (StS) writhe**: we numerically compute the global or non-local crossing number by

$$\omega_{StS}(i,j) = \frac{2}{4\pi} \sum_{k=i-\frac{l_w}{2}}^{i+\frac{l_w}{2}} \sum_{l=j-\frac{l_w}{2}}^{j+\frac{l_w}{2}} \frac{(\boldsymbol{t}_{k,k+1} \times \boldsymbol{t}_{l,l+1}) \cdot (\boldsymbol{r}_k - \boldsymbol{r}_l)}{|\boldsymbol{r}_k - \boldsymbol{r}_l|^3} \quad (9)$$

which measures the entanglement of a polymer segment centred at bead i against the segment centred at bead j. The window l_w is taken to be the persistence length $l = 10\sigma$ for most simulations.

Physically, Eqs. (7)-(9) effectively compute the average number of times the contiguous (for 1D) and non-contiguous (for 3D) segments of the polymer display crossings when observed from many different directions. A graphical and detailed description of these representations can be found in Ref. [10]. Below, we also refer to these quantities as "geometric representations" and they naturally lend themselves to be used as input features for neural networks because they are invariant under translations and rotations of the conformation and under relabelling of the beads.

This is by no means an exhaustive list of geometric features, and others have been implemented in the past for instance looking at pairwise distances between neighbouring or distant points along the knotted curves [11–13]. Yet, we argue that distances between segments are insensitive to the sign of the entanglement and will, as shown in the main text, yield less accurate ML models, as found with the unsigned writhe (see Fig. 1 in the main text).

KNOTS SHARING TOPOLOGICAL INVARIANTS

Among the knots considered in this work, many share one or more topological invariants. In Table I we summarise the couples/triplets of knots up to 10 crossings that display the same Alexander polynomial, for informational purpose.

9_8	8_{14}	10_{131}	10_{135}	10_{34}
9_{46}	6_1		10_{133}	7_6
9_2	7_4		10_{132}	5_1
9_{29}	9_{28}	10_{163}	10_{130}	7_5
9_{24}	8_{18}		10_{129}	8_8
10_{165}	9_{15}		10_{103}	10_{40}
10_{164}	10_{10}		10_{98}	10_{87}
10_{162}	10_{20}		10_{77}	10_{65}
10_{156}	8_{16}		10_{68}	10_{31}
10_{155}	8_9		10_{63}	9_{38}
10_{150}	10_{127}		10_{59}	9_{40}
10_{149}	9_{20}		10_{56}	10_{25}
10_{147}	8_{11}		10_{54}	10_{12}
10_{143}	8_{10}		10_{52}	10_{23}
10_{141}	8_5		10_{37}	10_{28}
10_{140}	8_{20}		10_{24}	10_{18}
10_{136}	8_{21}		10_1	8_3

TABLE I. Table summarising pairs or triplets of knots sharing the same Alexander polynomial.

NEURAL NETWORKS & TRAINING

The number of neurons comprising the input layer was determined according to the type of knot representation; the Cartesian coordinate and adjacent monomer distance representations used 3 neurons (one for each dimension) per bead, totalling 300 input neurons for $N = 100$ beads polymers, whilst most other geometric representations used one neuron per monomer, totalling N input neurons. The StS writhe representation used $N \times N$ input neurons.

Architecture optimisation

The optimal number of hidden layers, hidden units and other hyperparameters (including learning rate and batch size) for the FFNN were determined via an automated hyperparameter tuning software provided by Keras, known as *KerasTuner* [14], utilising an optimised random-grid search method with adaptive resource allocation and early stopping [15]. This scheme randomly selects a set of hyperparameters satisfying predefined ranges, builds the corresponding NN architecture and trains the model with the input representation for a set number of epochs before testing. This procedure is repeated for a given number of repetitions to find the best performing model and architecture. This hyperparameter tuning was achieved using the XYZ representation.

The hyperparameters obtained from this procedure are reported in Table II.

Hyperparameter	Value
Batch Size	256
N. hidden layers	4
N. hidden neurons in layer	320
Activation function	ReLU
Learning rate	10^{-3}
Weight Initialisation	Xavier Uniform Initialiser
Optimisers	Adam
Loss function	Sparse Categorical Cross-Entropy

TABLE II. A summary of the hyperparameters used for the FFNN using *KerasTuner*. The total number of trainable parameters is roughly $4\,10^5$.

Conversely, the RNN architecture was built without hyperparameter tuning, and we roughly matched the hyperparameters reported in Ref. [12] and summarised in Table III. We note that in contrast with Ref. [12] we chose the hidden layer activation function to be the hyperbolic tangent (tanh) as opposed to the ReLU function, as the TensorFlow GPU configuration was better optimised. We also used fewer number of hidden layers.

Following hyperparameter tuning, each knot feature representation was used to train, validate and test the performance of the different models on each task, resulting in a corresponding classification or localisation accuracy. For consistency, all NNs were trained for a maximum of 1000 epochs (though this limit was never reached, in practice), before utilising the testing set. These classification scores were then compared to determine which knot representation was optimal. Early stopping was used to prevent over/under fitting the NNs to the training data, improving generalisation and making training more computationally efficient [16]. In our case, early stopping meant training was halted when the validation error did not improve by a minimum of 0.001 over the course of 10 epochs; at this point, the weights producing the lowest validation error were restored for the testing phase. The NNs were implemented via the deep learning Python library TensorFlow with Keras 2.0 back-end [17] as this provided a simple and standardised framework for supervised learning tasks, with many highly optimised ML classes readily accessible.

Hyperparameter	Value
Batch Size	256
N. hidden layers	4 (2nd bidirectional)
N. hidden neurons in layer	100
Activation function	Hyperbolic Tangent
Learning rate	10^{-5}
Weight Initialisation	Xavier Uniform Initialiser
Optimisers	Adam
Loss function	Sparse Categorical Cross-Entropy

TABLE III. A summary of the hyperparameters considered in this work for the RNN model to match the ones in Ref. [12]. The total number of trainable parameters is roughly $4 \cdot 10^5$.

Dataset Splitting

In each task, our datasets were firstly partitioned into a stratified training-validation-testing split of 72%-18%-10%, respectively (chosen to match that selected by Vandans et al. [12]). For larger datasets, it was more efficient to adopt the splitting 90%-2.5%-7.5% to reduce the I/O load on the hardware during the validation step. This stratified split randomly allocates conformations to each set whilst maintaining equal proportions of each knot type, ensuring each split is equally representative of the knots being classified [18].

To prove that the splitting choice does not affect the final result, we considered three different splits of the 5 class dataset and trained/tested using such configurations. In Figure S1, results from the testing procedure indicate that accuracy is largely unaffected by the splitting choice; we therefore decided to adopt the choice with the largest training dataset to improve network robustness.

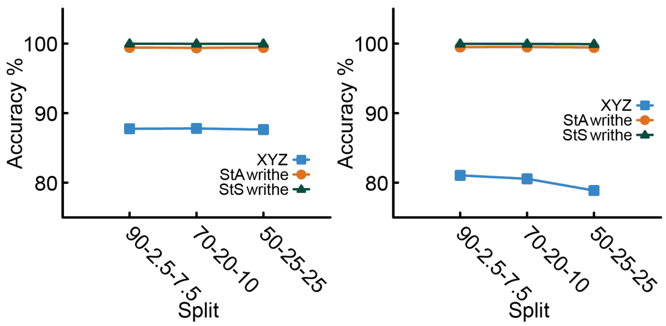

FIG. S1. Accuracy versus choice of split for RNN (left) and FFNN (right), respectively. The three different splits used for this comparison are reported on the x-axis.

Convergence

Convergence time depends on the input dataset and the feature used, as it is related to early stopping conditions and to how easily a NN can learn from a certain input feature. We noticed that for all the cases considered, training using XYZ coordinates takes many more epochs to converge, as opposed to training with StA or StS (Fig. S2). In fact, the number of epochs needed to train the NN with the StA and StS are minimally affected by the size of the classification problem. In Fig. S2 we plot, from left to right, training accuracy versus epochs for a 5 ($\leq$ 5 crossings knots), 8 ($\leq$ 6 crossing knots) and 15 ($\leq$ 7 crossing knots) class problem.

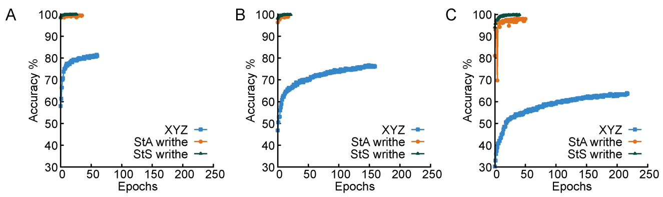

FIG. S2. Validation accuracy versus training epoch for a FFNN, from left to right up to 5 (A), 6 (B) and 7 (C) crossing knots datasets.

Effects of flexibility on network accuracy

It is reasonable to assume that the flexibility of the polymers in the dataset may affect the final training accuracy. This is because stiffer polymers may display fewer non-essential self-crossings than flexible ones. To test how robust our NN approach is with respect to curve flexibility, we generated new datasets using polymers with $l_p = 1$ and $l_p = 5$ for the 5-knots problem. We observed a sharp decrease in accuracy for most of the features and model architectures, in particular for XYZ-trained models (Fig. S3). We also implemented a different ML algorithm using a random forest model to compare with our neural network approaches. This model – with 300 trees and up to $4 \cdot 10^6$ nodes – dropped from 98% ($l_p = 10\sigma$) to $\sim$ 70% ($l_p = 1\sigma$) accuracy when trained on StA or StS writhe features, suggesting that this architecture is sensitive to small scale fluctuations of the polymer contour. At the same time, and irrespective of the precise NN architecture employed, XYZ coordinates yielded the poorest accuracy across the board (Fig. S3). In marked contrast to this, we observed that for both RNNs and FFNNs, training with StS writhe consistently returned an extremely high accuracy (>99%) for any persistence length investigated, strongly supporting the strength of this geometric feature over others.

Effects of increasing complexity on network accuracy

To test the robustness of our approach as a function of knot complexity (rather than size of classification problem), we choose four different combinations of 5 knots with increasing minimum crossing number and test the accuracy of the NNs on these different problems. More specifically, we consider these problems: (P1) 0_1, 3_1, 4_1, 5_1, 5_2 (P2) 5_1, 5_2, 6_1, 6_2, 6_3 (P3) 5_1, 5_2, 7_1, 7_2, 7_3 (P4) 9_1, 9_{42}, 10_1, 10_2, 10_{71}, which have increasing average minimum crossing number (or complexity) from around 3 to roughly 10. Notably, the last problem contains some knots that share the same HOMFLY polynomials. We provide confusion matrices and accuracies for these problems, tackled by FFNN with different input features in Fig. S4. Interestingly, the StS and StA features maintain an accuracy above 98% across the four problems. At the same time, and strikingly, the XYZ-trained NN yields more accurate predictions for higher complexity problems although remain well below the writhe-trained models.

Testing trained networks on longer knots

To further prove the generality of our features and models, we tested a network previously trained on knots with contour length $N = 100$ by feeding it features computed on knots with contour length $N = 200$. More specifically, we considered the StA writhe feature and binned it by summing adjacent beads to generate a 100-beads long StA signal. Testing our StA-trained NN on such data returned an accuracy of 89.9% (compared with a 95% accuracy score obtained by training the network directly on these 200σ long configurations), demonstrating that the network is robust enough to yield a reasonable accuracy even when given knots of longer length. Note that many LSTMs can be trained using a zero-padding method to accept data of variable input size.

This strategy could be used to make our pre-trained NNs more accurate in handling polymers of different lengths.

Additionally, we tested the robustness of the StS writhe feature by training a FFNN network on a 5 class classification problem with $N = 200$ beads long polymers. In Fig. S5 one can appreciate that training the FFNN with the StS writhe feature retains a very high accuracy even with longer polymers, while the StA writhe and XYZ trained NNs display a significant reduction in accuracy.

Testing trained networks on never seen knots

We have also tested our trained FFNNs on never-seen knots. Specifically, we tried to classify a 6_1 (twist) and 7_1 (torus) knot using 5-class XYZ and StA trained networks. The results is that the StA is better than XYZ at identifying the geometric family of the knot, so for instance the 6_1 is more frequently classified as closest twist knot (4_1) by the StA than the XYZ feature. Similarly, the StA convincigly classifies the 7_1 as the closest torus knot 5_1. These results are similar to the ones obtained in Ref. [13] (see Fig. S6). The fact that StA-trained networks predict a 4_1 knot when fed with a 6_1 may imply that these models give more importance to the pattern of entanglements rather than the absolute number of crossings. Oppostely, XYZ-trained models appear to give more importance to the total number of crossings irrespectively of their orientation.

Principal Component Analysis

In order to better understand the relationship between different knots and how these are represented in the XYZ and local writhe spaces, we have performed principal component analysis (PCA) on 1000 configurations of the first 5 simplest knots. As expected, the (COM-subtracted) XYZ coordinates of the knots all cluster together in the reduced PC space, and display reduced spreading due to the topological constraints on more complex knots (Fig. S7). On the contrary, the StA writhe features, originally living in a 100-dimensional space (one value per bead index) are clearly separated in the reduced 2D PCA space (Fig. S7). One key observation is that the distributions could be clustered even with a single PCA dimension and that the pair of knots whose StA distributions partially overlap are the 0_1 and 4_1 because their **global** writhe is the the same and is zero. Global writhe is not unique for different knots and so we expect a large degree of co-clustering when more complex knots are included in the analysis. Finally, StA features identify a 100x100 dimensional space which is then less efficiently clustered in separate regions in the reduced 2D PCA plot (Fig. S7).

Robustness of models trained with StA features computed using different window lengths

There is a natural length scale for the window length l_w to be used when computing the StA and StS writhe that is the scale of polymer fluctuations. Effectively, our unit length of self-entanglement has to be the persistence length l_p. Taking window length much bigger than the persistence length leads to smoothing of entanglement features (see also Ref. [19], Appendix C, Fig. 12). Nonetheless, we repeated the 5 class training of our ML models with different window lengths and – surprisingly – the accuracy is very robust (see Fig. S8). We find that it decreases significantly only when the window length equals the whole polymer length, in which case StA is constant and equals the global writhe. In this case, we find that, as expected, the knots 0_1 and 4_1 are confused with each other as they have the same global writhe, i.e. zero [20] (see Fig. S8). In general, we thus expect that as long as $l_w < N$ the ML models will be able to use StA and StS writhe to efficiently distinguish knots. Instead, when $l_w \simeq N$, the accuracy will become worse when more complex knots with similar global writhe are included in the training.

Localisation of knots on open curves

In this section we have attempted the localisation of knots on open curves. More specifically, we used the RNN model trained at localising knots within closed curves and asked if it could detect knotted segments within an open curve initially prepared as a 5_1 knot. The model can detect the shortest knotted portion of the curve even on open curves on which it was not trained on (see Fig.S9). The model trained on StA features is, once again, more accurate at identifying the shortest knotted contour. Interestingly, notice the last snapshot before unknotting is completely missed by the XYZ-trained model (predicted knotting probability P_k is constant and below 0.5), while the StA-trained network can correctly identify a short knot, as identified through kymoknot.

---

* joint first author
† corresponding author, davide.michieletto@ed.ac.uk

[1] K. Kremer and G. S. Grest, J. Chem. Phys. **92**, 5057 (1990).
[2] S. Plimpton, J. Comp. Phys. **117**, 1 (1995).
[3] L. Tubiana, E. Orlandini, and C. Micheletti, Phys. Rev. Lett. **107**, 1 (2011).
[4] L. Tubiana, G. Polles, E. Orlandini, and C. Micheletti, European Physical Journal E **41**, 1 (2018).
[5] A. Stasiak, V. Katritch, J. Bednar, D. Michoud, and J. Dubochet, Nature **384**, 122 (1996).

[6] D. Michieletto, Soft Matter **12**, 9485 (2016).

[7] K. Klenin and J. Langowski, Biopolymers **54**, 307 (2000).

[8] J. Smrek, J. Garamella, R. Robertson-Anderson, and D. Michieletto, Science Advances **7**, 1 (2021).

[9] M. Baiesi, E. Orlandini, F. Seno, and A. Trovato, Journal of Physics A: Mathematical and Theoretical **50** (2017), 10.1088/1751-8121/aa97e7.

[10] J. L. Sleiman, R. H. Burton, M. Caraglio, Y. A. Gutierrez Fosado, and D. Michieletto, ACS Polym. Au **2**, 341 (2022).

[11] P. Johanns, P. Grandgeorge, C. Baek, T. G. Sano, J. H. Maddocks, and P. M. Reis, Extreme Mechanics Letters **43**, 101172 (2021).

[12] O. Vandans, K. Yang, Z. Wu, and L. Dai, Physical Review E **101**, 1 (2020).

[13] A. Braghetto, S. Kundu, M. Baiesi, and E. Orlandini, Macromolecules **56**, 2899 (2023), arXiv:2212.11822.

[14] T. O'Malley, E. Bursztein, J. Long, F. Chollet, H. Jin, L. Invernizzi, *et al.*, "Keras Tuner," `https://github.com/keras-team/keras-tuner` (2019).

[15] L. Li, K. Jamieson, G. DeSalvo, A. Rostamizadeh, and A. Talwalkar, Journal of Machine Learning Research **18**, 1 (2018).

[16] Y. Yao, L. Rosasco, and A. Caponnetto, Constructive Approximation **26**, 289 (2007).

[17] M. Abadi, A. Agarwal, P. Barham, E. Brevdo, Z. Chen, C. Citro, G. S. Corrado, A. Davis, J. Dean, M. Devin, S. Ghemawat, I. Goodfellow, A. Harp, G. Irving, M. Isard, Y. Jia, R. Jozefowicz, L. Kaiser, M. Kudlur, J. Levenberg, D. Mané, R. Monga, S. Moore, D. Murray, C. Olah, M. Schuster, J. Shlens, B. Steiner, I. Sutskever, K. Talwar, P. Tucker, V. Vanhoucke, V. Vasudevan, F. Viégas, O. Vinyals, P. Warden, M. Wattenberg, M. Wicke, Y. Yu, and X. Zheng, "TensorFlow: Large-scale machine learning on heterogeneous systems," (2015), software available from tensorflow.org.

[18] K. Sechidis, G. Tsoumakas, and I. Vlahavas, in *Machine Learning and Knowledge Discovery in Databases*, edited by D. Gunopulos, T. Hofmann, D. Malerba, and M. Vazirgiannis (Springer Berlin Heidelberg, Berlin, Heidelberg, 2011) pp. 145–158.

[19] D. Michieletto, Soft Matter **12**, 9485 (2016).

[20] A. Stasiak, V. Katritch, and L. Kauffman, *Ideal Knots*, K & E series on knots and everything (World Scientific, 1998).

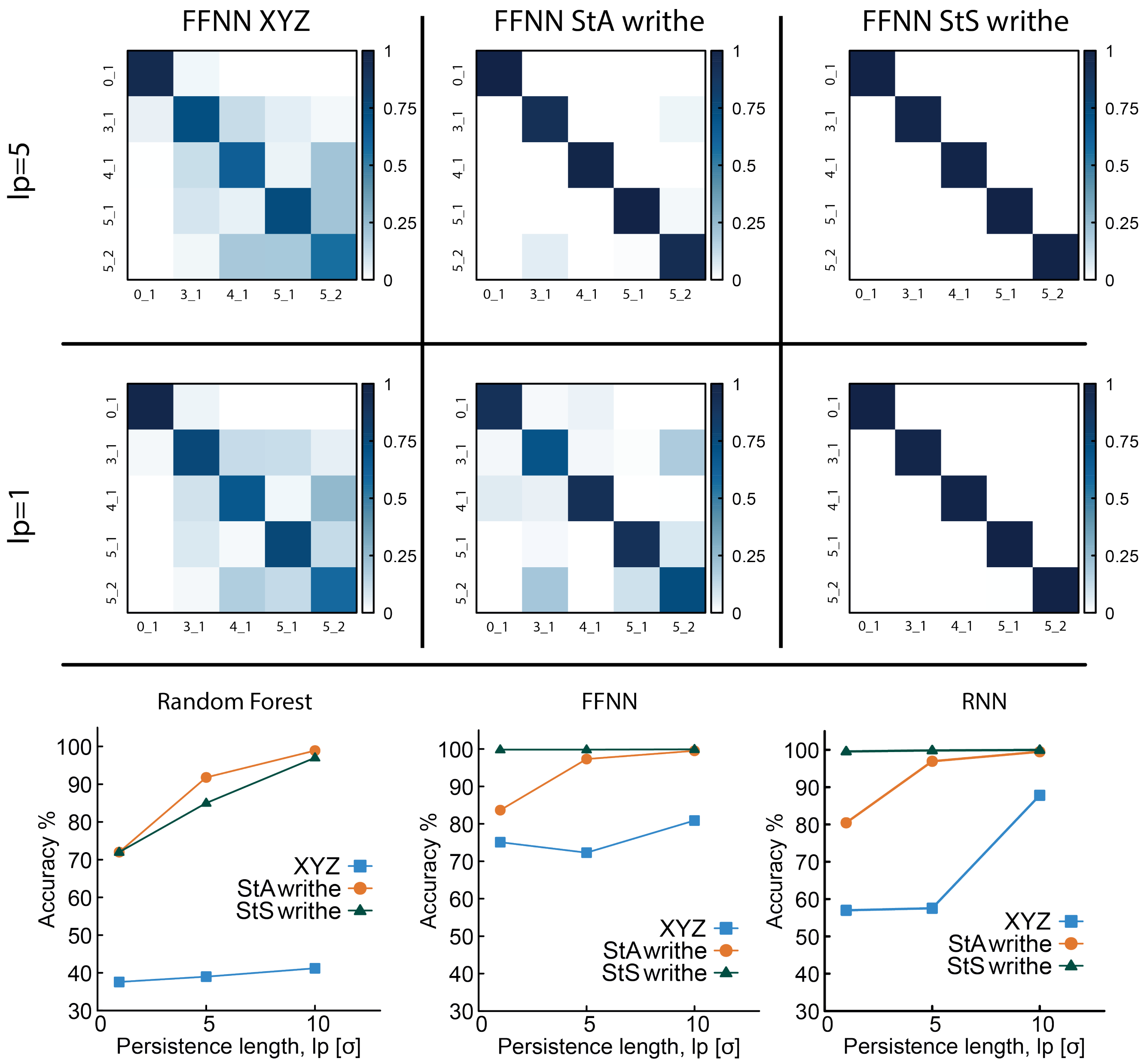

FIG. S3. Confusion matrices for XYZ, StA and StS writhe input features (from left to right) for datasets generated using different persistence lengths, $l_p = 1\sigma$ and $l_p = 5\sigma$ (the case $l_p = 10\sigma$ is reported in the main text) for a 5-class classification problem. At the bottom, we plot the accuracy versus the persistence length for the different input features and different architectures (random forest, FFNN and RNN).

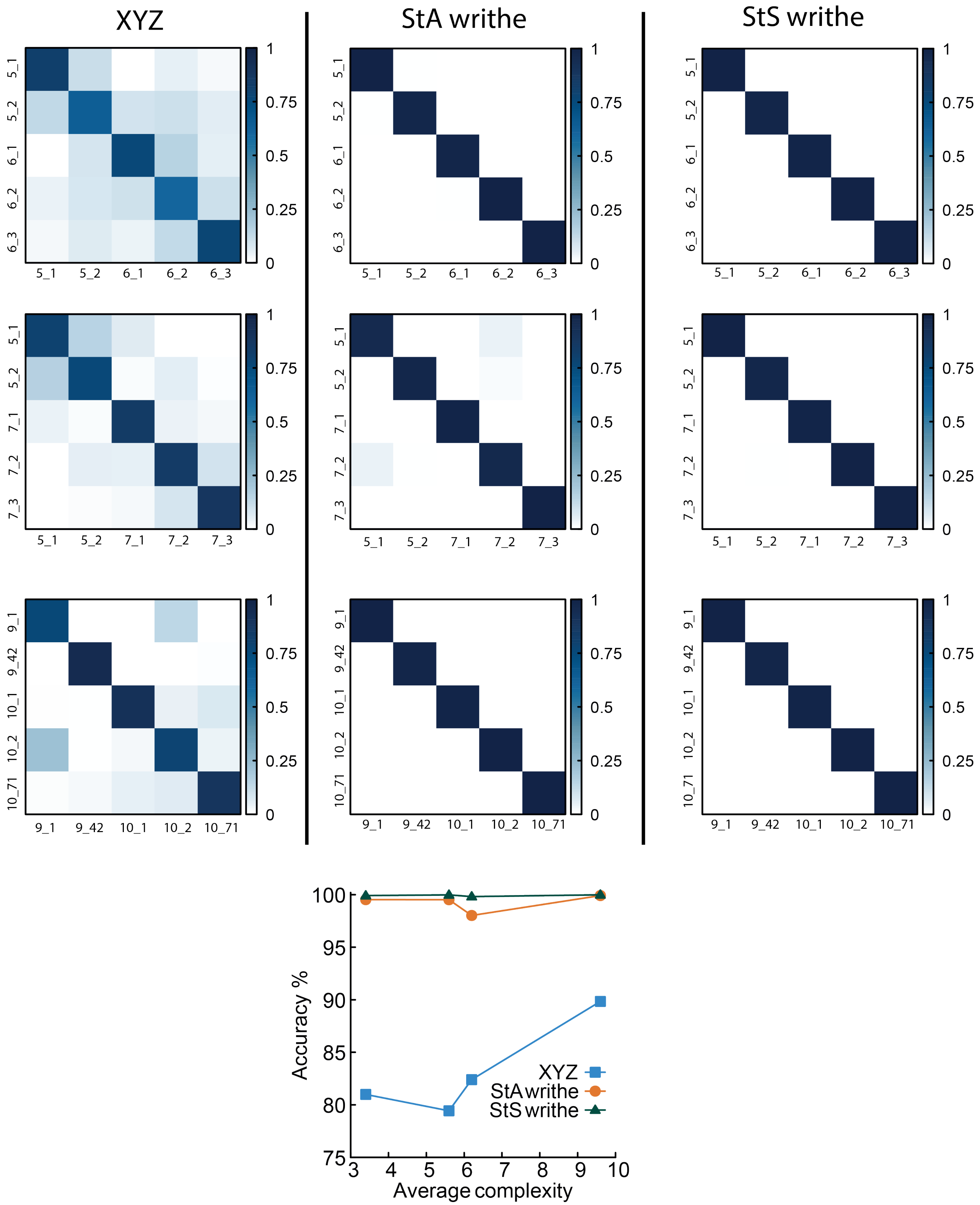

FIG. S4. Confusion matrices for XYZ, StA and StS writhe input features (from left to right) for different combinations of knots in a 5-class classification problem. At the bottom, we plot the accuracy versus the average knot complexity, defined as the average minimal crossing number of the knots considered in each problem. The precise knot types in each problem are reported on the confusion matrices.

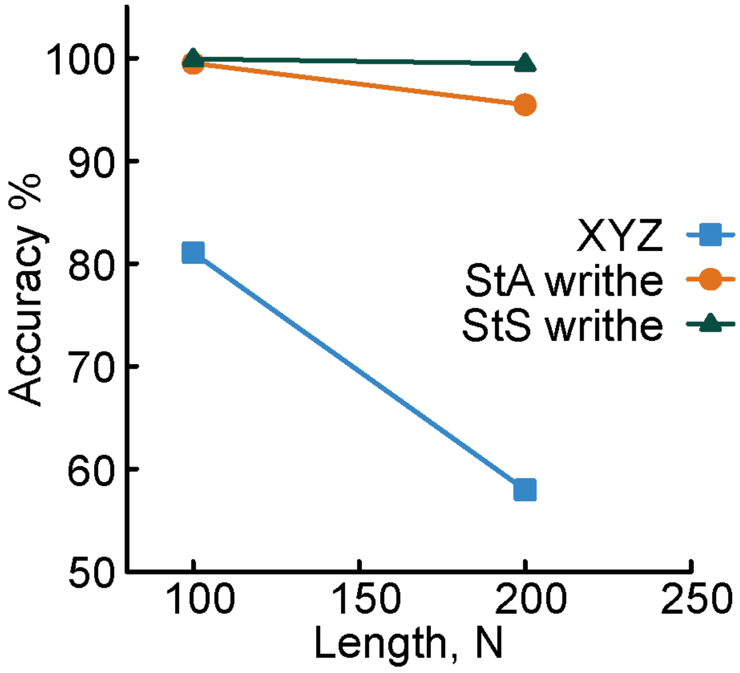

FIG. S5. Accuracy versus knot length N, for a FFNN trained to classify the 5 simplest knots using different input features. The StS representation remains robustly close to 100% accuracy (99.5%) even on longer polymers.

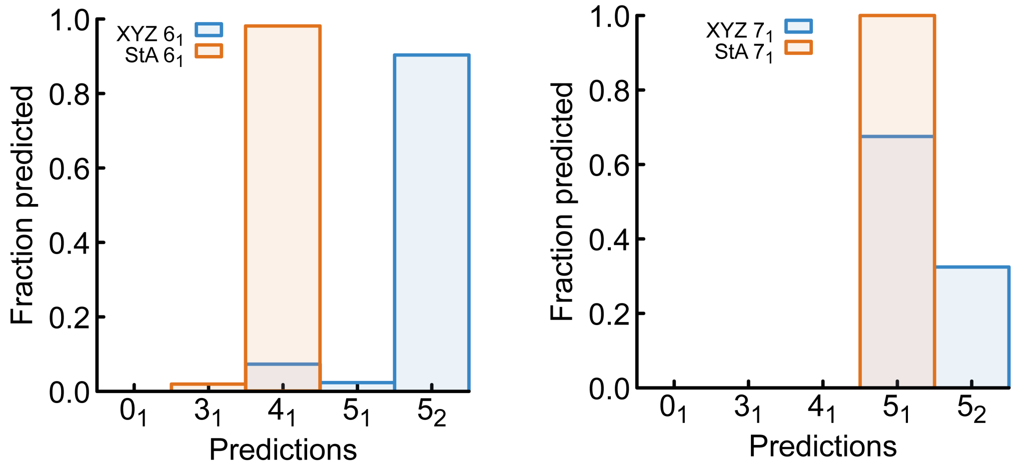

FIG. S6. Fraction of predicted knots, over 7500 instances of 6_1 or 7_1 knots never seen by a 5-class XYZ or StA model. The 6_1 is classified either as a 5_2 or as 4_1 (both twist knots) by the XYZ and StA trained NNs respectively. The 7_1 is convincingly classified as a 5_1, the closest torus knot.

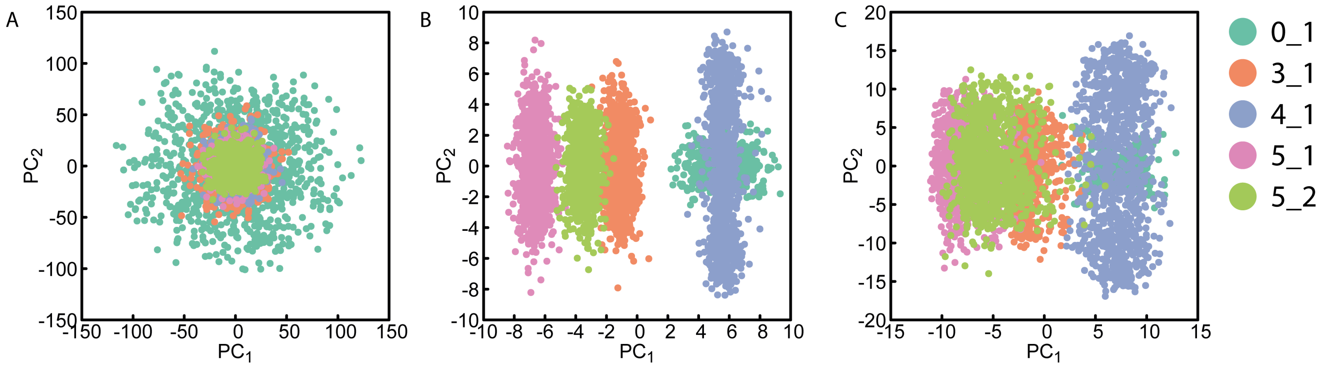

FIG. S7. From left to right: PCA for XYZ, StA and StS features.

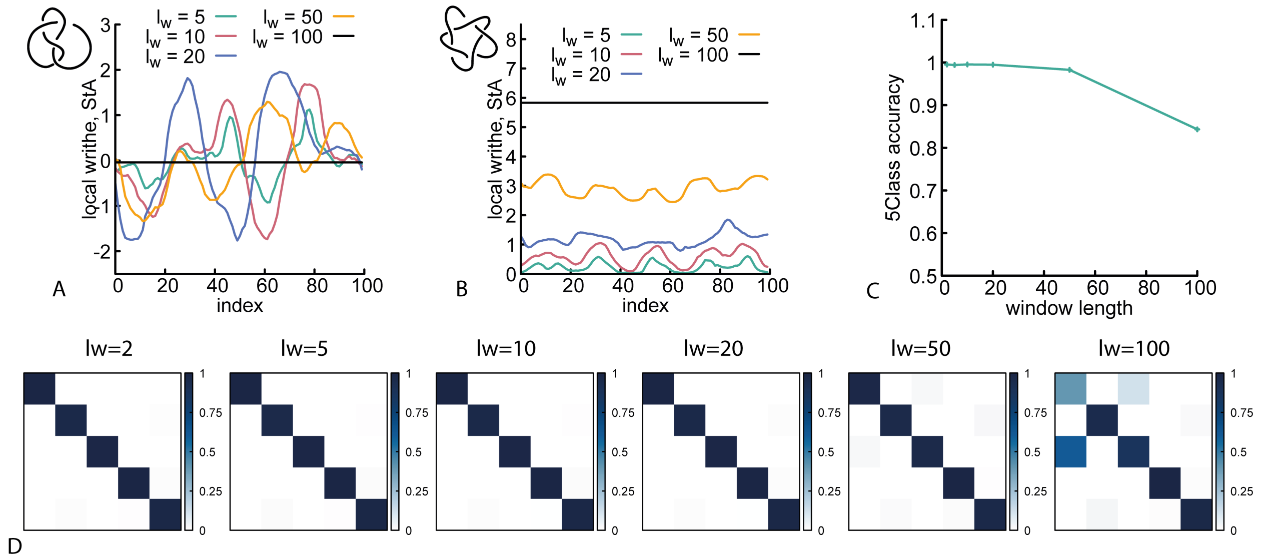

FIG. S8. **A** StA writhe for the 4_1 using different window lengths. **B** StA writhe for the 5_1 using different window lengths. **C** Accuracy of the models as a function of window length. **D** Confusion matrices for the 5 class classification problem for different window lengths.

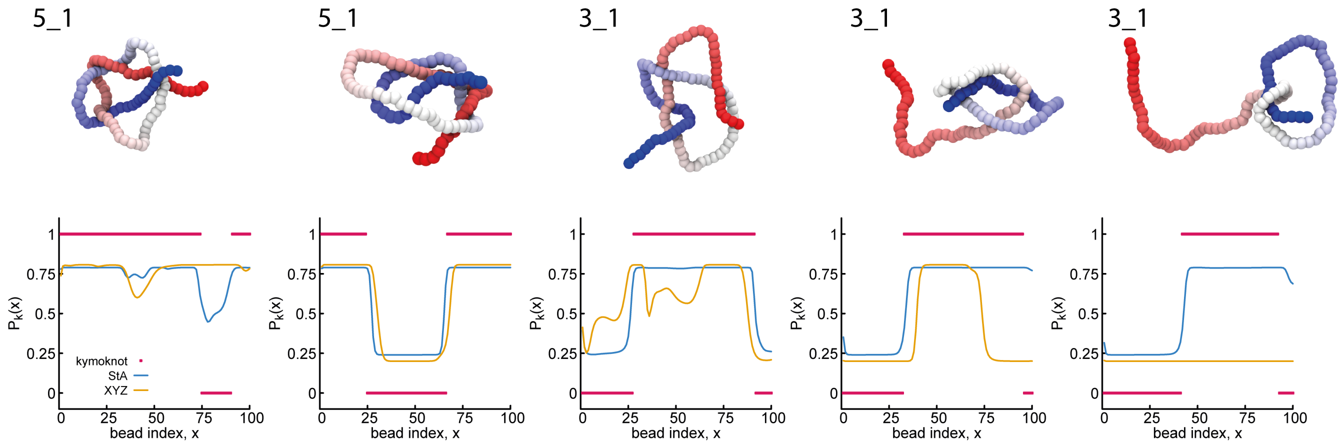

FIG. S9. From left to right the undoing of a 5_1 knot to simpler topologies. The unknotting stages and location of shortest knotted arcs are well tracked by the localisation RNN model trained with StA features.
